# SHR1032, a novel STING agonist, stimulates anti-tumor immunity and directly induces AML apoptosis

**DOI:** 10.1038/s41598-022-12449-1

**Published:** 2022-05-20

**Authors:** Chunying Song, Dong Liu, Suxing Liu, Di Li, Ivana Horecny, Xinzhu Zhang, Puhui Li, Lei Chen, Matthew Miller, Rasheduzzaman Chowdhury, Mena Issa, Ru Shen, Yinfa Yan, Fengqi Zhang, Lei Zhang, Limin Zhang, Chang Bai, Jun Feng, Linghang Zhuang, Rumin Zhang, Jing Li, Hilary Wilkinson, Jian Liu, Weikang Tao

**Affiliations:** 1 Eternity Bioscience Inc., 6 Cedarbrook Drive, Cranbury, NJ 08512 USA; 2 Shanghai Hengrui Pharmaceutical Co. Ltd., 279 Wenjing Road, Shanghai, 200245 China

**Keywords:** Cancer, Drug discovery, Immunology

## Abstract

Stimulator of interferon genes (STING) activation induces type I interferons and pro-inflammatory cytokines which stimulate tumor antigen cross presentation and the adaptive immune responses against tumor. The first-generation of STING agonists, cyclic di-nucleotide (CDN), mimicked the endogenous STING ligand cyclic guanosine monophosphate adenosine monophosphate, and displayed limited clinical efficacy. Here we report the discovery of SHR1032, a novel small molecule non-CDN STING agonist. Compared to the clinical CDN STING agonist ADU-S100, SHR1032 has much higher activity in human cells with different STING haplotypes and robustly induces interferon β (IFNβ) production. When dosed intratumorally, SHR1032 induced strong anti-tumor effects in the MC38 murine syngeneic tumor model. Pharmacodynamic studies showed induction of IFNβ, tumor necrosis factor α (TNFα) and interleukin-6 (IL-6) in the tumors and, to a lower extent, in the plasma. More importantly, we found SHR1032 directly causes cell death in acute myeloid leukemia (AML) cells. In conclusion, our findings demonstrate that in addition to their established ability to boost anti-tumor immune responses, STING agonists can directly eradicate AML cells, and SHR1032 may present a new and promising therapeutic agent for cancer patients.

## Introduction

Stimulator of interferon genes (STING) is a transmembrane protein located in the endoplasmic reticulum (ER). In eukaryotic cells, the DNA-sensing nucleotidyl transferase enzyme cyclic GMP-AMP synthase (cGAS) directly binds to cytosolic DNA from bacterial pathogens, some DNA viruses or under certain conditions mammalian DNA itself. The activated cGAS converts GTP and ATP into 2ʹ, 3ʹ-cyclic guanosine monophosphate–adenosine monophosphate (cGAMP), which binds to STING, leading to its conformational changes and trafficking from the endoplasmic reticulum (ER) to the Golgi to perinuclear endosomes. Consequently, STING recruits tank-binding kinase 1 (TBK1), which phosphorylates IFN regulatory factor 3 (IRF3), leading to its translocation into the nucleus to drive the transcriptional induction of type I interferons and the nuclear factor-κB (NF-κB)-dependent expression of proinflammatory cytokines^1^.

Besides its predominant role in microbial infection, STING signaling also carries out other evolutionarily conserved functions, which can be mechanistically and functionally separated from interferons^2^. For instance, STING-mediated signaling has a conserved role in autophagy^3^. Some recent studies reported STING activation in T cells leads to cell death^4,5^.

Type I interferons selectively stimulate cross-presentation of tumor antigens and mobilization of tumor-specific CD8 T cells, which primes the adaptive immune response against tumors. Immune checkpoint inhibitors have demonstrated clinical efficacy for multiple tumor types. However, a significant portion of tumors are cold or non-inflamed, which have a limited T cell infiltrate and are not responding to immune-checkpoint therapies. This could potentially be overcome by stimulation of innate immune cells within the tumor microenvironment to enhance adaptive immunity. It has been shown in preclinical mouse tumor models that STING agonists can activate innate and adaptive immunity, leading to increased T cell infiltration and superior anti-tumor activity^6^.

The ability of STING activation to generate anti-tumor activity in cold tumors makes it an attractive cancer therapeutic target. However, the first generation of STING agonist, cyclic di-nucleotide (CDN), showed limited anti-tumor efficacy in clinical studies^7–9^. One potential mechanism underlying the disconnection between clinical and preclinical efficacy is the differential activities of STING agonists against human STING and mice STING. The poor permeability and low in vivo stability might also contribute to the limited clinical efficacy of CDN STING agonists. Many pharmaceutical companies are working on non-CDN STING agonists to overcome the limitations of the first generation CDN compounds^10–12^.

Here we report the discovery of a novel non-CDN small molecule, SHR1032, which is a strong agonist against human STING. Despite its relatively weak in vitro activity against mouse STING, it induced IFNβ production and displayed significant tumor growth inhibition when delivered intratumorally in a MC38 murine syngeneic tumor model. In addition, we found STING activation can directly and strongly induce interferon-independent cell death in human AML cells. Our studies indicate this novel small molecule STING agonist may provide a new therapeutic agent for the treatment of solid tumors and AML.

## Results

### Identification of a novel chemical scaffold with STING agonistic activity

**Figure 1:**
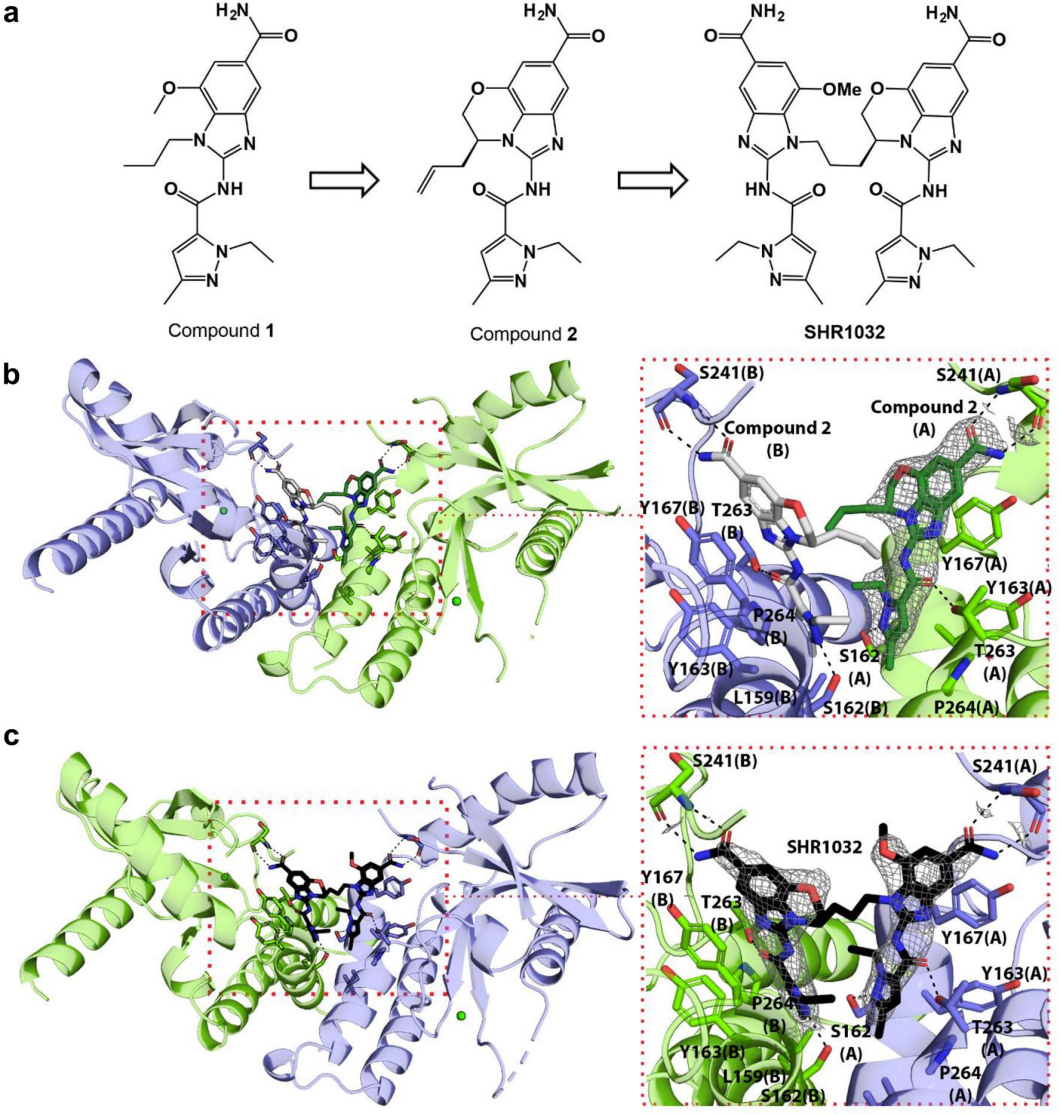
Discovery of SHR1032 and views from the crystal structures of STING in complex with compound 2 and SHR1032. (**a**) Scheme showing identification of GSK compound 1 (GSK patent)-derived compound 2 that led to SHR1032. Figures (**b**) and (**c**) compare views from crystal structures of compound 2-STING and SHR1032-STING complexes (overview in left and close-up view in right panels). STING monomers (A and B) are color-coded in green and blue. Electron densities around the ligands (grey meshes representing difference Fo-Fc maps) are contoured to ~ 3σ. The structures revealed overall similar binding modes of the monomeric compound 2 and the dimeric SHR1032 to STING with that of a previously reported structurally similar amidobenzimidazole (diABZI) agonist (PDB ID: 6DXL)^10^. Like the symmetric diABZI dimer, the asymmetric SHR1032 (grey sticks) binds in the cGAMP binding site that is located at the dimeric interface of STING (**c**). The 1-ethyl-3-methyl-1H-pyrazole-5-carboxamide moiety of each SHR1032 monomer binds at the hydrophobic cleft formed by the side chains of L159, S162, Y163, T263, and P264 of both STING monomers. In addition, the structure reveals H-bond interactions (dotted lines) between pyrazole nitrogen of SHR1032 and the hydroxyl group of S162, and between the carboxamide of SHR1032 with T263. The aromatic ring of the 1H-benzo[d]imidazole moiety forms hydrophobic interaction with Y167, while the terminal amide forms a H-bond network with S241. There is no apparent interaction between the SHR1032 C3-linker and the protein, resulting in a loss of clear electron density around the linker, nor there is any obvious interaction involving the OMe group.

We sought to develop small molecule compounds with increased STING agonist activity against human STING. GSK reported a series of non-CDN small molecules, represented by compound 1^13^, with good human STING agonistic activity. Based on our molecular modeling analysis, along with the reported CDN-STING crystal structures, we designed a novel series of fused tricyclic heterocycle compounds and analyzed their binding to human STING with thermal shift assays and the potency in activation of STING signaling with a reporter cellular assay (Supplementary Table S1). Thermal shift assays measure the difference between apo STING and ligand bound STING in the inflection point at which 50% of protein is thermally unfolded (ΔTm). THP1 is a human monocytic cell line derived from an acute monocytic leukemia patient and has been routinely used to study the cGAS/STING pathway as they induce a type I IFN response to cGAMP and dsDNA that is cGAS/STING dependent^14^. The THP1 reporter cells stably express a secreted Lucia luciferase reporter gene controlled by five IFN-response elements. The ability of compounds to activate the IRF reporter gene in the THP1 cells was also evaluated. We explored various substitutes on the morpholine ring^15^ and identified compound 2 with good binding affinity for human STING protein (ΔTm = 5.7 °C) and THP1 reporter cellular potency (EC50 = 1.6 µM) (Fig. 1a and Supplementary Table S1).

We initially obtained a crystal structure of compound 2 in complex with human STING (Fig. 1b). Although the complex was crystallized in C2221 space group with a single molecule per asymmetric unit, analysis of the buried surface area and complementarity between two symmetry related molecules suggested that this assembly likely represented a biologically relevant dimer. The structure revealed two molecules of compound 2 bound in the cGAMP binding pocket per STING dimer that are in proximity and raised the possibility that covalently linking two such monomers might produce dimeric compounds with higher potency as previously observed^10^. After exploring combinations of different monomers and various linkers, we identified compound SHR1032 (Fig. 1a; Supplementary Table S1) with significantly improved binding to STING and cellular reporter assay (thermal shift ∆Tm = 10.7 °C; EC_50_ = 0.03 µM). We then obtained the crystal structure of SHR1032 complexed with human STING protein (Fig. 1c) in C2 symmetry with 2 molecules per asymmetric unit that validated our predicted binding mode in the cGAMP binding pocket of STING. The structure revealed Fo-Fc difference density for a single SHR1032 that spanned between the two monomers resulting in a loss of higher C2221 symmetry. Details of the key interactions between SHR1032 and the protein dimer are described in Fig. 1.

### SHR1032 displays high potency towards major human STING alleles and activates cGAS-STING signaling

**Figure 2:**
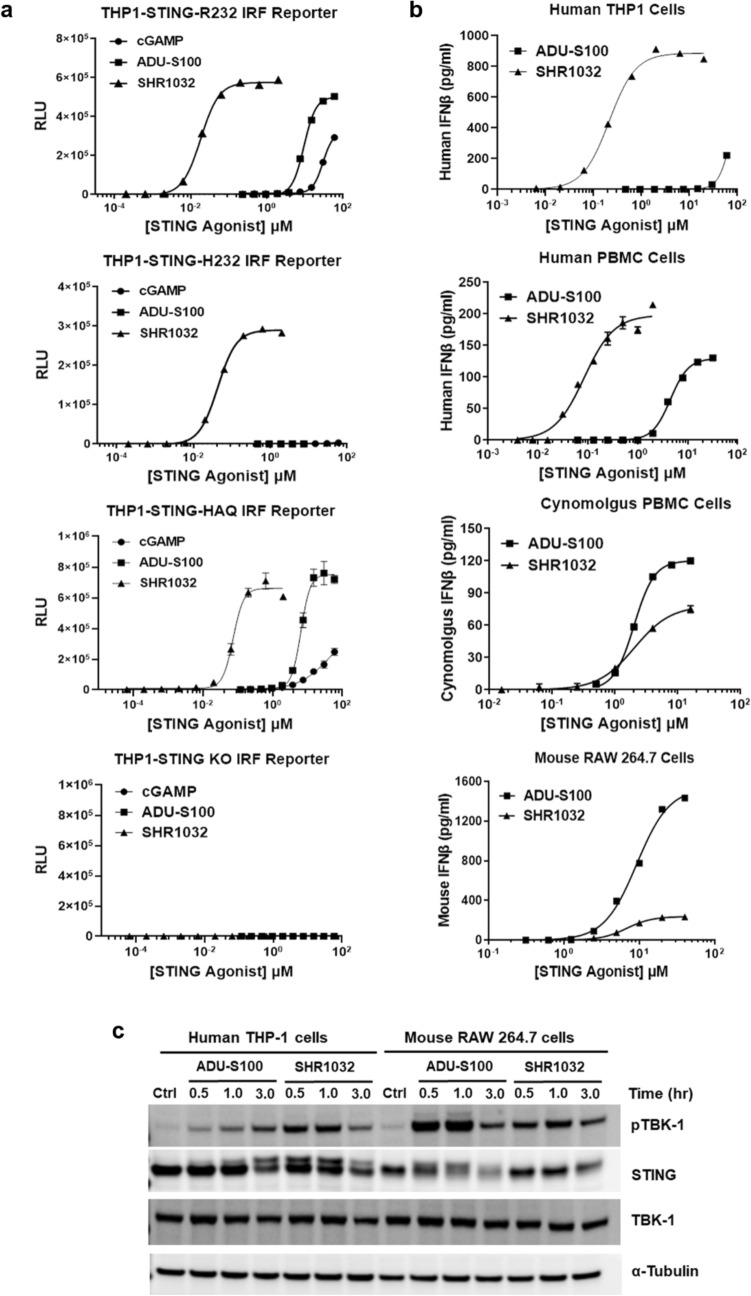
SHR1032 specifically and potently activates STING signaling pathway. IRF luciferase reporter assays in (**a**) THP1-STING-R232, THP1-STING-H232, THP1-STING-HAQ, and THP1-STING-KO cells. Cells were stimulated for 24 h with the indicated STING compounds and assessed for luciferase activity. IFNβ production and activation of cGAS-STING signaling pathway in (**b**) human THP1-STING-R232, human PBMCs from a donor homozygous for the R232 variant, cynomolgus PBMC, and mouse RAW164.7 macrophage cells were treated with STING agonists for 5 h. IFNβ in media was quantified by human or mouse ELISA kits respectively. (**c**) THP1-STING-R232 cells and mouse RAW164.7 macrophage cells were stimulated with 40 µM of ADU-S100 or 10 µM of SHR1032 for the indicated time points. The amount of pTBK1, total TBK1, STING and α-tubulin was measured by western blotting.

Five haplotypes of human STING have been identified (R232, H232, HAQ, AQ, and Q alleles), which vary at amino acid positions 71, 230, 232, and 293^16^. R232, HAQ and H232 were reported to be the three major alleles. We tested the responsiveness of the human STING alleles to STING agonists in THP1 reporter cells carrying human STING R232, H232 and HAQ variants. Compared to endogenous STING agonist cGAMP and the clinical compound ADU-S100, SHR1032 has much better potency in all the three reporter cell lines tested (Fig. 2a). To examine whether the activities of STING agonists are on-target, we treated THP1-STING-KO reporter cells with the agonists and no luciferase reporter activity was detected, confirming the cellular activity of STING agonists we observed are specific from STING.

To further determine whether SHR1032 activates downstream STING signaling, we assessed THP1-STING-R232 reporter cells for induction of IFNβ by ELISA (Fig. 2b). Consistent with the luciferase reporter activation, much higher level of IFNβ was induced by SHR1032 than that of ADU-S100. To examine the activation of STING signaling in primary human cells, we stimulated human PBMCs and measured the production of human IFNβ (Fig. 2b). SHR1032 induced much greater amount of human IFNβ than ADU-S100. Some STING agonists were reported to have differential activities across species^17^. We assessed agonistic activities of SHR1032 in cynomolgus PBMC cells and mouse RAW164.7 macrophage cells (Fig. 2b). The monkey and mouse IFNβ production with SHR1032 stimulation was lower than that with that of ADU-S100, but still detectable. The on-target pathway activation was validated by the phosphorylation of TBK1 with Western blot analysis in THP1-STING-R232 and RAW164.7 mouse macrophage cells treated with SHR1032 and ADU-S100 at the time points indicated, respectively (Fig. 2c). Consistent with their human and mouse activities in IFNβ induction, SHR1032 induced a higher level of TBK1 phosphorylation than ADU-S100 in THP1 cells; whereas in mouse RAW164.7 macrophage cells, TBK1 phosphorylation induced by SHR1032 is lower. STING activation correlated with an increase in the apparent molecular weight of STING, which has been reported to be due to its phosphorylation upon activation^18^. These results from cell-based assays indicate that SHR1032, a non-CDN small molecule agonist, is capable of activating STING pathway in human cells harboring different STING alleles.

### SHR1032 induces strong in vivo anti-tumor immunity

**Figure 3:**
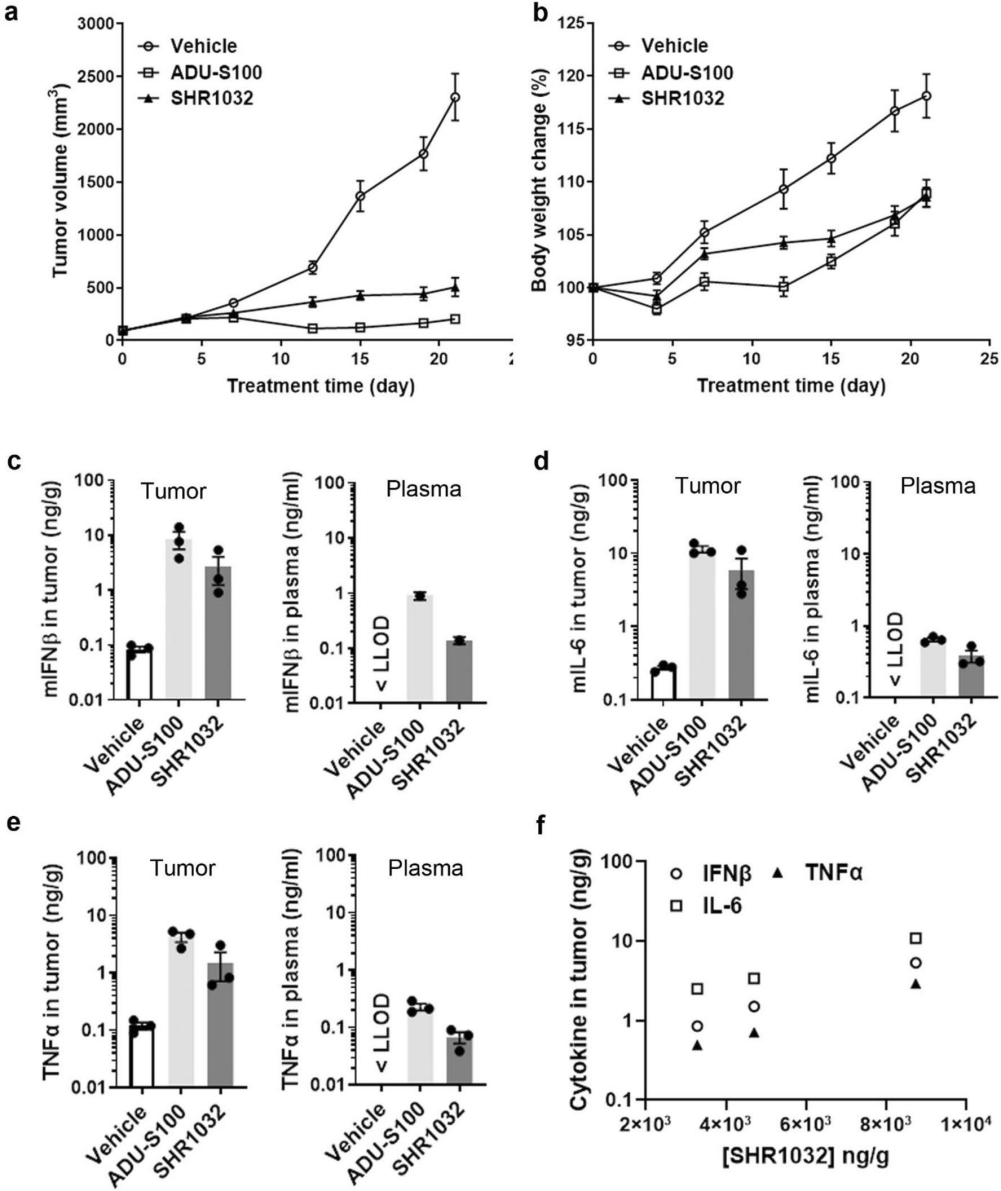
Robust cytokine induction and antitumor effects in vivo. (**a**,**b**) C57BL/6 mice were inoculated with mouse MC38 colon cancer cells in the left flank. When tumor volumes were 100 mm^3^, they received intratumoral (IT) dose of 100 µg of SHR1032 on day 1, 5, 8 and 12 or IT dose of 50 µg ADU-S100 on day 1, 5, and 8, or vehicle, respectively. (**a**) Tumor volume was measured at different time points. Results are shown as mean tumor volume ± sem. (n = 8 animals per group). (**b**) Mouse bodyweight was measured at different time points. (**c**–**f**) C57BL/6 mice were inoculated with mouse MC38 colon cancer cells in the left flank. When tumor volumes were 100 mm^3^, they received one IT dose of 100 µg of SHR1032 or 50 µg ADU-S100, or vehicle. (**c**) IFNβ, (**d**) IL-6 and (**e**) TNFα from both tumor and plasma was measured by ELISA 4 h after. (**f**) SHR1032 compound concentration in tumors were measured. Cytokine levels and compound concentration shows good correlation.

To evaluate whether SHR1032 could augment anti-tumor immunity in vivo, tumor growth inhibition was determined in the MC38 mouse syngeneic colorectal adenocarcinoma models using WT C57BL/6 mouse. Following the published study^19^, 50 µg of ADU-S100 was injected intratumorally per tumor each day on day 1, day 5, day 8, respectively. Since SHR1032 displayed weaker mouse activity in vitro than ADU-S100, 100 µg was delivered 4 doses intratumorally on day 1, day 5, day 8 and day 12, respectively. Consistent with the published results^19^, ADU-S100 induced complete tumor regression after 21 days since the first dosing. Interestingly, even with a weaker mouse activity, SHR1032 induced 78% of tumor growth inhibition as compared to the control group (Fig. 3a). Both STING agonists treatment didn’t lead to animal body weight change indicating there was no overt toxicity effects (Fig. 3b). The anti-tumor activity in this mouse model was unlikely due to direct killing of MC38 cells by STING activation since STING agonists had no effect on MC38 cell viability as assessed by in vitro Cell TiterGlo assays (Supplementary Fig. S1).

To evaluate pharmacodynamic markers for target engagement in vivo, IFNβ, IL-6 and TNFα protein concentrations in tumor and circulating plasma collected 4 h post the initial dosing were measured (Fig. 3c–e). SHR1032 induced all the three cytokines in both tumors and plasma. Moreover, SHR1032 displayed favorable mouse pharmacokinetic properties. Compound concentrations are correlated well with cytokine levels in tumors from different mice (Fig. 3f). The above results suggested SHR1032 can induce immunity to suppress tumor growth and may have greater anti-tumor immunity in human due to its better human activity.

### STING activation directly inhibits AML cell growth

**Figure 4:**
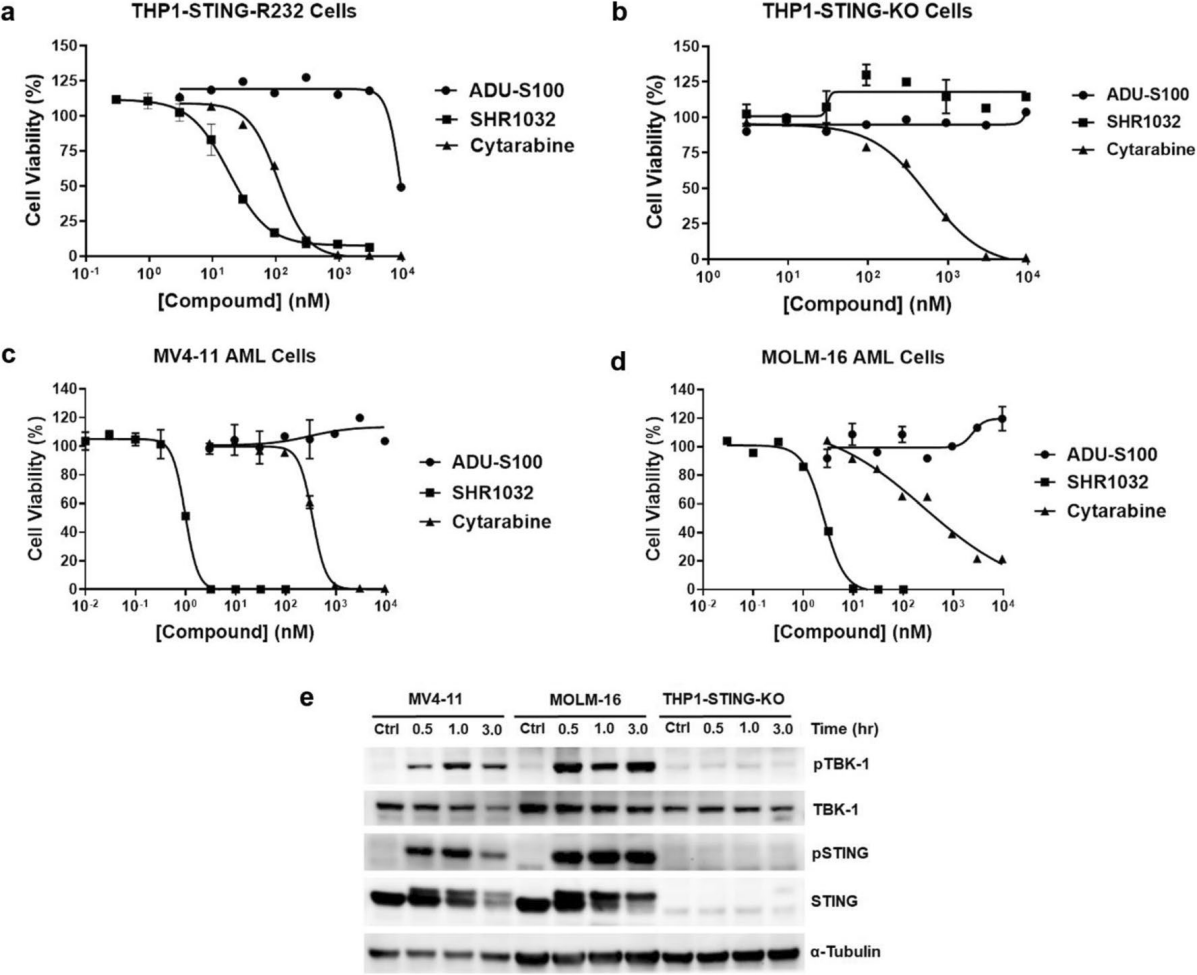
SHR1032 inhibits AML cell growth and displays specific activation of STING signaling pathway in AML cell lines. (**a**–**d**) AML cells were treated with STING agonists and standard of care cytarabine, and cell viability was measured by CellTiter Glo in (**a**) THP1-STING-R232, (**b**) THP1-STING-KO, (**c**) MV-4-11 and (**d**) MOLM-16. (**e**) MV-4-11, MOLM-16 and THP1-STING-KO cells were stimulated with 1 µM of SHR1032 for the indicated time points. The amount of pTBK1, total TBK1, pSTING, total STING and α-tubulin was measured by Western blot. *pSTING* phosphorylated STING.

During our studies of STING agonists, we serendipitously observed STING activation reduced THP1 cell viability. As shown in Fig. 4a, SHR1032 suppressed THP1-STING-R232 cell growth (GI_50_ = 23 nM) even more effectively than cytarabine (GI_50_ = 100 nM), which is the drug used as standard of care for AML. ADU-S100 showed a weaker cell growth inhibition effect (GI_50_ = 9441 nM) than SHR1032, which correlates with its lower potency than SHR1032 in STING activation. Next, we wanted to investigate whether this antiproliferative effect from STING agonists was due to off-target effect. The results showed that the cell growth was not detectable in THP1-STING-KO cells with SHR1032 or ADU-S100 treatment (Fig. 4b), while cytarabine had similar growth inhibitions in STING-WT and -KO cells, suggesting that the growth inhibition was on-target or STING-specific.

Next, we searched the public databases for STING expression level in AML. We found AML cell lines (including MV4-11 and MOLM-16) have the highest STING expression level compared to other cancer cell lines in the CCLE database (https://depmap.org/portal/gene/STING). More importantly, STING expression is higher in primary AML tumor cells than paired normal control cells (http://gepia.cancer-pku.cn/). This led us to test more cell lines to verify the hypothesis that SHR1032 and ADU-S100 may selectively inhibit AML cell viability. By Sanger sequencing, we found MV4-11 and MOLM-16 carry STING R232/H232 and H232/H232 alleles (Supplementary Table S2), which were shown to respond to SHR1032 readily (Fig. 2a). Interestingly, MV4-11 and MOLM-16 cells are very sensitive to SHR1032 treatment. The GI_50_s of SHR1032 (1.0 nM and 2.6 nM, respectively) are much lower than the GI_50_s of cytarabine (340 nM and 450 nM, respectively) in both cell lines.

To validate the on-target pathway activation, the phosphorylation of TBK1 and STING were examined with Western blot analysis in MV4-11, MOLM-16 cells. THP1-STING-KO cells were used as a negative control. As expected, SHR1032 induced TBK1 and STING phosphorylation in both MV4-11 and MOLM-16 cells, but not in THP1-STING-KO cells at the time points indicated (Fig. 4e). Activation caused an upward shift on SDS-PAGE and led to double bands in total STING Western blots due to phosphorylation. Together, the above results showed that AML has high STING expression and suggested that SHR1032 activation of STING results in significant AML cell growth inhibition.

### STING activation induces apoptosis in AML cells

**Figure 5:**
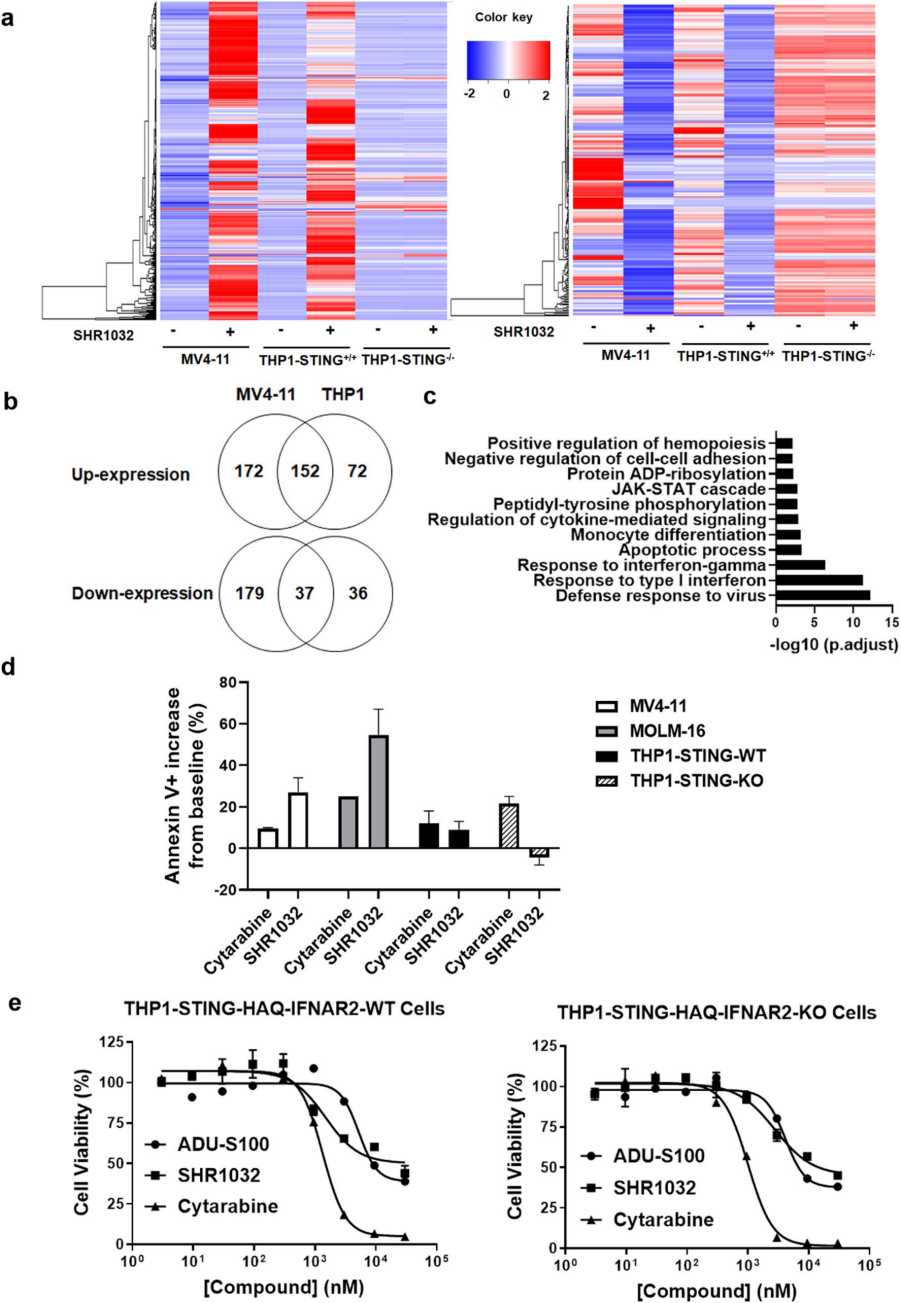
Cell apoptosis induced by SHR1032 in AML cell lines is independent of type I interferon. (**a**–**d**) RNA-Seq analysis of THP1-STING-R232, THP1-STING-KO and MV4-11 AML cells treated with 1 µM of SHR1032 for 4 h. (**a**) Heatmaps showing gene expression patterns of upregulated (left) or downregulated (right) DEGs with SHR1032 treatment. (**b**) Venn diagram showing genes up- (above) or down-regulated (below) genes in MV4-11 and THP1-STING-R232 cells with SHR1032 treatment. (**c**) Gene Ontology analysis of those genes up-regulated in both MV4-11 and THP1-STING-R232 cells. (**d**) ANNEXIN V staining analysis with flow cytometry in MV4-11, MOLM-16, THP1-STING-WT and THP1-STING-KO cells. (**e**) Cell viability analysis by CellTiter Glo in THP1-IRFAR2-WT or -KO cells.

Many early studies described the role of STING in antiviral gene expression, and a few studies reported STING-activated cell death in T cells^4,5,20^. To understand how cell death is regulated by STING activation in the AML cells, we took an unbiased approach to identify genes regulated by STING activation in both MV4-11 and THP1 cell lines using THP1-STING-KO cell line as a negative control. The cells were treated with or without SHR1032 for 4 h, and the differentially expressed genes (DEGs) for all the three cell lines were analyzed by RNA-sequencing. The patterns of up- and down-regulated DEGs from each pairwise comparison are shown in heat maps (Fig. 5a; left, up; right, down). We found 324 and 216 genes were up- and down-regulated with SHR1032 treatment in MV4-11 cells respectively, and 224 and 73 genes were up- and -downregulated respectively in THP1-STING-WT cells, while no gene expression was significantly changed in THP1-STING-KO cells (Supplementary Table S3). This validates the selectivity of SHR1032 for STING. Further analysis of the DEGs showed that 152 and 37 genes are up- or down-regulated in both MV4-11 and THP1-STING-WT cells (Fig. 5b). The up-expressed genes in both cell lines (SHR1032 treatment versus DMSO-treatment) were enriched for distinct Gene Ontology (GO) terms (Fig. 5c). Not surprisingly, most enriched processes are related to virus defense responses and IFN signaling. Interestingly, activation of cysteine-type endopeptidase activity involved in apoptotic process is found to be enriched as well. The genes upregulated in this apoptotic process are CASP10, JAK2, TNFSF10, HIP1R, RIPK1, PML, and NOXA. Together, these results indicate that SHR1032 induces de novo apoptotic genes leading to cell death in AML cells.

Next, we determined whether STING activation leads to the apoptosis of AML cells using Annexin V staining quantified by flow cytometry. Compared to cytarabine, SHR1032 induced a much higher level of apoptosis in MV4-11 and MOLM-16 cells, and a comparable level of apoptosis in THP1 cells (Fig. 5d). While cytarabine induced similar levels of apoptosis in THP1-STING-WT and THP1-STING-KO cells, SHR1032 had no effect in the STING knockout cells, indicating its effect on apoptosis is STING-dependent. These results correlate with the growth inhibition in CellTiter Glo analysis in the same three cell lines (Fig. 4a–d).

Type I interferons have been known to drive apoptosis in AML cells^21^. Therefore, the apoptotic effects might be caused by type I interferons generated by STING activation. To address this question, we examined the IFNβ production with ELISA in MV4-11 and MOLM-16 cells stimulated with SHR1032. Both cell lines produced IFNβ, but with a level much lower than that in THP1 cells (supplementary Fig. S2), indicating no correlation exists between apoptosis and IFN. The activity of type I IFNs is mediated by the IFN-α receptor (IFNAR), which consists of two chains: IFNAR1 and IFNAR2. Knockout of the IFNAR2 gene abolishes the cellular response to type I IFNs. To determine whether STING activation induced death is dependent on type I IFNs, we compared cell viability levels in THP1-IFNAR2-WT and THP1-IFNAR2-KO cells with Cell TiterGlo (Fig. 5e) and found IFNA receptor deletion has no effect on cell death induced by SHR1032. Thus, our findings suggest that STING activation directly induces AML cell death through transcriptional regulation of apoptosis genes.

## Discussion

The cGAS-STING pathway is a key regulator of innate immune sensing of cancer, with a strong potential to enhance tumor rejection through the induction of adaptive immune responses mediated predominantly by type I interferons. Its immune-modulatory properties have made the STING pathway a promising target for cancer immunotherapy. Despite remarkable efficacy in many pre-clinical studies, only modest effects have been reported from clinical trials of CDN STING agonists^7,8^. Suboptimal potency for human STING agonists and poor pharmacokinetic properties might be the key underlining reasons.

We have discovered a novel series of non-CDN small molecule STING agonists with fused tricyclic core via structure-based drug design (SBDD). Further rational optimization based on the information from the cocrystal structures (Fig. 1b,c) and structural activity relationship (SAR) led to the identification of SHR1032, which has greatly increased human STING agonistic activity compared to the CDN compounds. It exhibits selective and potent activity across all known major isoforms of the human STING protein. R232/R232, R232/H232 and R232/HAQ isoforms of STING protein cover more than 89% of the European population^22^. SHR1032 readily activates the STING/TBK1/IRF3 pathway and induces the expression of type I IFN in cells in a STING-dependent manner. Intermittent intratumoral dosing produces potent and durable anti-tumor immunity in mice bearing syngeneic tumors. The high potency for human STING along with the impressive preclinical efficacy supports the further development of SHR1032 as a potential immunotherapy approach for cancer patients.

Immune stimulation is commonly recognized as the main signaling activity of STING. However, STING also triggers other biological processes. One important physiological role of STING signaling is autophagy, which is a more ancient activity and operates independently of TBK1 and IRF3^3,23^. In addition, several studies have shown STING agonists could induce ER stress and cell death in mouse T-lymphocytes^4,5,20^. It was reported that the magnitude of STING signaling determines the outcomes as mouse T cells which show high STING expression, but not mouse macrophages, which show low STING expression, die of apoptosis upon STING activation^20^. Tang et al. reported that STING activation triggers mitochondria-mediated apoptosis in normal and malignant mouse B cells^24^. Although it has been clearly shown that STING agonists could induce cell death in mouse lymphocytes, whether they could directly induce cell death in other cell types, especially human cancer cells, is of interest for further investigation. Acute myeloid leukemia (AML) is a malignancy of immature myeloid cells. Chemotherapeutics are the current standard-of-care with limited efficacy. To our knowledge, this is the first study that demonstrates that STING activation in human AML cells induces cell death, which might be related to the high expression level of STING (CCLE; http://gepia.cancer-pku.cn/) or specific pathway(s) in AML cells. SHR1032 shows a much higher potency than cytarabine, the standard-of-care, in killing human AML cells. With the more specific activity of STING signaling, STING agonists, such as SHR1032, may be superior therapeutic options in safety and efficacy to cytarabine for AML therapy.

Type-I IFNs induce AML cell death in preclinical studies and the use of IFNα for clinical treatment of AML dates to the early 1980s^21^. Intriguingly, our studies demonstrate that even though STING activation stimulates type-I IFN production in AML cells, the cell death that is induced is not dependent on type-I IFN signaling. Our gene profiling analysis indicates STING activation might induce apoptosis of AML cells through de novo gene expression.

## Materials and methods

### Cells

Human monocyte THP-1 Dual cells (InvivoGen), THP1-Dual™ KO-STING Cells (InvivoGen) and THP1-Dual KI-hSTING-R232 cells (InvivoGen) were cultured in RPMI 1640, 2 mM L-glutamine, 25 mM HEPES, 10% heat-inactivated fetal bovine serum, 100 μg/ml Normocin, Pen-Strep, and 10 µg/ml of blasticidin, 100 μg/ml of Zeocin. Mouse RAW 264.7 cells (ATCC) were cultured in DMEM with 10% heat-inactivated fetal bovine serum and Pen-Strep. MV4-11 cells (ATCC) were cultured in ATCC-formulated Iscove's Modified Dulbecco's Medium with 10% heat-inactivated fetal bovine serum and Pen-Strep. MOLM-16 cells (DSMZ) were cultured in RPMI 1640 with 20% heat-inactivated fetal bovine serum. Frozen human peripheral blood mononuclear cells (PBMCs) and Cynomolgous monkey PBMCs were purchased from Stem Cell Technologies and iQ Biosciences respectively.

### Thermal shift assay

The melting temperature (Tm) of human STING was determined by calculating the temperature at the inflection point of the thermal melt curve generated on a Roche LightCycler 480 using 384 plate format and 465–590 nm filter set. Samples were prepared by combining protein at a final concentration of 5 uM with ligands at 100, 50, and 10 µM, followed by the addition of 5 × concentration SYPRO Orange dye. Plates were centrifuged briefly and incubated for 5 min at room temperature. Florescence was measured while ramping the plate temperature from 20 to 95 °C and the resulting data was processed using ROCHE data analysis software. Delta Tms were calculated by subtracting the Tm of untreated protein from ligand treated samples. The DMSO concentration was kept constant for all ligand concentrations and control measurements.

### THP1-dual reporter assay, cell growth quantification and western blotting

THP1 reporter cells were treated for 24 h with a total of 100 k cells and 150 ul media in each well a 96-well plate. Following the incubation, 20 µl of supernatant was transferred to an opaque 96 well ½ area plate and incubated for 30 s with 50 µl of QUANTI-Luc substrate (InvivoGen) per manufacturer’s instructions. Luminescence signal was measured using Tecan infinite M1000. EC50 values were calculated using non-linear regression analysis of GraphPad Prism. Cell growth was monitored by CellTiter-Glo Luminescent Cell Viability Assay (Promega) per manufacture’s protocol. GI50 values were calculated using non-linear regression analysis of GraphPad Prism. Cell lysates for western blotting were made in RIPA buffer (Sigma) with Halt Protease and Phosphatase Inhibitors (Thermo Scientific). Antibodies against pSTING, TBK1, pTBK1 and tubulin were purchased from Cell Signaling Technologies, and anti-STING was purchased from Protein Tech.

### Cell staining and flow cytometric analysis

Cells were incubated with compounds or DMSO for 16 h in a 6-well plate (0.5 × 10^6^ cells/well in 2 ml of media). After incubation, cells were collected and washed once in stain buffer (BD Biosciences). Samples were stained with FITC_Annexin V (BioLegend) for 15 min at room temperature. Then the cells were washed twice and resuspended in 200 µl stain buffer, and analyzed with Guava EasyCyte (Millipore).

### ELISA assays

Human and Cynomolgous PBMC’s were thawed and spun down in Lymphocyte media (ZENBIO) and resuspended in media to 1.5 x 10^6^ cells/ml density. Mouse RAW 264.7 cells were detached from the flask and resuspended in media to 1.5 x 10^6^ cells/ml density. Then, 100 µl of media containing the cells was added to each well of a 96-well plate containing compound in 50 µl of media. Plates were incubated for 5 h at 37 °C and supernatant was collected for ELISA analysis. Human and monkey IFN-β protein concentration was measured using human IFN-β Quantikine ELISA Kits (R&D Systems) per manufacture’s protocol. Mouse IFN-β was measured using mouse IFN-β Quantikine ELISA Kits (R&D Systems) per manufacture’s protocol. Mouse TNFα, IFN-β and IL-6 in the tumor lysates and plasma was measured by Quantikine ELISA Kits (R&D Systems) per manufacture’s protocol.

### PCR-sanger sequencing

Genomic DNA was isolated from cells using DNeasy Blood and Tissue Kit (Qiagen). STING variants of the cell lines were determined with PCR-Sanger sequencing by GeneWiz.

### RNA-seq and data analysis

RNA was extracted from cells using RNeasy Plus Mini Kit (Qiagen). Library preparation with NEBNext Ultra II with PolyA selection, sequencing with NovaSeq and data analysis was performed by Admera Health, New Jersey. The De-Seq2 (version 1.14.1) was used to do the differential analysis. Gene expression levels were quantified as Fragments Per Kilobase Million (FPKM). Gene expression with FPKM value ≥ 0.5 were selected and Log2-transformed for the Differential Gene Expression (DEGs) analysis using iDEP (http://bioinformatics.sdstate.edu/idep/). DEGs were defined by p < 0.05 within each indicated paired-comparison and then used for Gene Ontology Analysis and heatmap generation. The Gene Ontology Analysis was done using Enrichr. The heatmap was created using R package gplots (version 3.1.1), function heatmap.2.

### Murine in vivo studies—MC38 syngeneic tumor model

Female C57BL/6 mice, aged between 8 and 10 weeks old, were purchased from Charles River Lab. MC38 mouse colorectal cancer cells were resuspended as single cell solution in PBS at a concentration of 5 × 10^6^ cells/ml. One hundred µl of cell suspension were implanted in the right flank of mice subcutaneously. Tumor size was measured by a caliper in three dimensions and its volume was then calculated according to the following formula: Tumor Volume (mm^3^) = l * w * h * 0.5236, where l = length, w = width and h = height in mm for a tumor. When the average tumor volume reached around 100 mm^3^, the mice were randomly assigned into three groups (n = 8) and each group of mice received one regimen. This day was assigned as day 0 for the study. SHR1032 was dosed at 100 µg in a volume of 50 µl via intratumor route per each animal on day 0, 4, 7, and 11; ADU-S100 was dosed at 50 µg in a volume of 50 µl via intratumor route per each animal on day 0, 4 and 7. Tumor volume and body weight were measured 3 times per week throughout the study. For pharmacodynamic biomarker studies, four hours post the first dosing on day 0, tumor and plasma samples were collected. Tumor samples were homogenized in PBS buffer with Halt Protease and Phosphatase Inhibitors (Thermo Scientific) and the supernatant was collected. Cytokines and SHR1032 concentration in the plasma and tumor tissues were analyzed. The in-life part of the study was conducted by Cephrim Biosciences, Inc. The experimental protocol used in this study was approved by the Institutional Animal Care and Use Committees (IACUC) of Cephrim Biosciences, Inc. The experiments were conducted in accordance with the Guiding Principles for the Care and Use of Laboratory Animals and complied with the ARRIVE guidelines.

### Protein production and crystallography

Human STING was produced and crystallized as described^10^. Purified recombinant human STING protein expressed in E. coli was concentrated to 10 mg/ml in 20 mM HEPES pH 7.5, 300 mM NaCl 10% Glycerol. Hanging drop crystallization drops were formed by adding 1 ul of protein solution with 1 ul of the well solution. The 24 well trays were filled with 1 ml well solutions consisting of a grid of 18–22% PEG 3350 verse 100 mM Tris buffer from pH 7 to 8. Crystallization trays were incubated at 18 °C for 24 h at which time crystals were transferred to a solution that was composed of the well solution with an additional 20% glycerol and varying concentrations of ligands. After a brief soak in the presence of the ligand the crystals were flash cooled in liquid nitrogen in preparation for data collection. Data were collected at 100 K using synchrotron radiation at the National Synchrotron Light Source II (NSLS-II) beamlines and were processed using CCP4 suite^25^. Structures were solved by molecular replacement using PHASER^26^ (search model PDB ID 6DXL) and refined using the maximum-likelihood function and bulk-solvent modelling. Parameter and topology files for compound 2 and SHR1032 were generated using PRODRG^27^. Iterative cycles of model building in COOT^28^ and refinement proceeded until the Rcryst/Rfree values converged. MOLPROBITY^29^ was used to monitor the geometric quality of the models between refinement cycles and identify poorly modelled areas needing attention. Water molecules were added to peaks > 1σ in 2Fo–Fc electron density maps that were within hydrogen bonding distance to protein with reasonable hydrogen bonding geometry and were refined. Data collection and refinement statistics are in Supplementary Table S4.

## Supplementary Information

Supplementary Information.

## Data Availability

All data needed to evaluate the conclusions in the paper are presented in the paper and the supplementary information S1.
