# Supplementary material for: SHR1032, a novel STING agonist, stimulates anti-tumor immunity and directly induces AML apoptosis
Source: Sci Rep. 2022 May 20;12:8579. doi: 10.1038/s41598-022-12449-1 (PMC9122897; doi:10.1038/s41598-022-12449-1)
Supplement: Supplementary file 1 — Supplementary Information. [file 41598_2022_12449_MOESM1_ESM.docx]

**Supplementary information**

**Table S1. STING binding and *in vitro* reporter analysis of compound 2 and SHR1032.**

| **Compound** | **Thermal shift ∆Tm (°C)** | **THP1 reporter cell EC_50_ (µM)** |
| --- | --- | --- |
| **Compound 2** | 5.7 | 1.6 |
| **SHR1032** | 10.7 | 0.03 |

**Table S2. Determination of STING alleles in THP1, MV4-11 and MOLM-16 cells by PCR-Sanger sequencing.**

| **Cell line** | **R71H** | **G230A** | **R232H** | **R293Q** |
| --- | --- | --- | --- | --- |
| **THP1** | H/H | A/A | R/R | Q/Q |
| **MV4-11** | R/R | G/G | R/H | R/R |
| **MOLM-16** | R/R | G/G | H/H | R/R |

| **Genes differentially expressed** | **MV4-11** | **THP1-STING-WT** | **THP1-STING-KO** |
| --- | --- | --- | --- |
| **Up-expressed** | 324 | 224 | 0 |
| **Down-expressed** | 216 | 73 | 0 |

**Table S3. Differentially expressed genes in MV4-11, THP1-STING-WT and THP1-STING-KO cells treated with or without SHR1032.**

**Table S4.** **Data collection and refinement statistics.**

|  | Compound 2 | SHR1032 |
| --- | --- | --- |
| **PDB acquisition codes** | 7T9U | 7T9V |
|  |  |  |
| **Data collection** |  |  |
| Space group | *C*222_1_ | C2 |
| Cell dimensions |  |  |
| *a*, *b*, *c* (Å) | 79.91, 89.39, 73.30 | 91.31, 80.88, 73.13 |
| α, β, γ (°) | 90.0, 90.0, 90.0 | 90, 89.99, 90.0 |
| Resolution (Å) | 59.56-2.46 (2.59-2.46)* | 73.13-2.68 (2.82-2.68)* |
| *R*_sym_ or *R*_merge_ | 0.219 (1.940)* | 0.110 (1.620)* |
| *I* / σ*I* | 7.30 (1.30)* | 9.40 (1.10)* |
| Completeness (%) | 99.9 (99.7)* | 94.9 (95.9)* |
| Redundancy | 13.4 (13.9)* | 7.3 (7.3)* |
|  |  |  |
| **Refinement** |  |  |
| Resolution (Å) | 39.95-2.46 | 60.55-2.68 |
| No. reflections | 9782 | 14248 |
| *R*_work_ / *R*_free_ | 0.224/ 0.233 | 0.205/ 0.235 |
| R.m.s. deviations |  |  |
| Bond lengths (Å) | 0.007 | 0.004 |
| Bond angles (°) | 1.294 | 0.820 |

*Highest resolution shell shown in parentheses.

**Figure S1. Cell viability analysis of MC38 cells with Cell TiterGo. MC38 cells were treated with SHR1032, ADU-S100 and Staurosporine for 72 hours with the concentrations indicated.**

**Figure S2. IFNβ measurement in MV4-11, MOLM-16 and THP1-STING-R232 cells treated with SHR1032 for 5 hrs.**

**Figure S3. Original images of Figure 2c.**

Two membrane blots were cut into 2 pieces guided by the marker to blot STING total, TBK-1-p, TBK-1 total and tubulin. Lane 1-7: THP1 cells. Lane 1, DMSO control; lane 2-4; treated with ADU-S100 for 0.5, 1 and 3 hrs respectively; lane 5-7, treated with SHR1032 for 0.5, 1 and 3 hrs respectively; lane 8-14: RAW264.7 cells. Lane 8, DMSO control; lane 9-11, treated with ADU-S100 for 0.5, 1 and 3 hrs respectively; lane 12-14: treated with SHR1032 for 0.5, 1 and 3 hrs respectively.

**STING-p**

marker 1 2 3 4 5 6 7 8 9 10 11 12 13 14

**α-Tubulin**

marker 1 2 3 4 5 6 7 8 9 10 11 12 13 14

**TBK-1-p**

marker 1 2 3 4 5 6 7 8 9 10 11 12 13 14

**TBK-1**

marker 1 2 3 4 5 6 7 8 9 10 11 12 13 14

**Figure S4. Original images of Figure 4e.**

Two membrane blots were cut into pieces guided by the marker to blot STING-p, STING total, TBK-1-p, TBK-1 total and tubulin. Lane 1-4: MV4-11 cells: 1, DMSO control; 2-4, treated with SHR1032 for 0.5, 1 and 3 hrs respectively. Lane 5-8: MOLM-16 cells: 1, DMSO control; 2-4, treated with SHR1032 for 0.5, 1 and 3 hrs respectively. Lane 9-12: THP1-STING-KO cells: 1, DMSO control; 2-4, treated with SHR1032 for 0.5, 1 and 3 hrs respectively.

**STING-p**

1 2 3 4 5 6 7 8 9 10 11 12 marker

**STING**

1 2 3 4 5 6 7 8 9 10 11 12 marker

**α-Tubulin**

1 2 3 4 5 6 7 8 9 10 11 12 marker

**TBK1-p**

1 2 3 4 5 6 7 8 9 10 11 12 marker

**TBK1**

1 2 3 4 5 6 7 8 9 10 11 12 marker
